# Supplementary material for: Spatial profiling of chromatin accessibility in mouse and human tissues
Source: Nature. 2022 Aug 17;609(7926):375–83. doi: 10.1038/s41586-022-05094-1 (PMC9452302; doi:10.1038/s41586-022-05094-1)
Supplement: Supplementary file 1 — Supplementary Figs. 1–12, statistics and reproducibility, the legends for Supplementary Tables 1–5 and Supplementary References. [file 41586_2022_5094_MOESM1_ESM.pdf]

nature portfolio

https://doi.org/10.1038/s41586-022-05094-1

Supplementary information

Spatial profiling of chromatin accessibility in mouse and human tissues

In the format provided by the
authors and unedited

Supplementary information

Spatial profiling of chromatin accessibility in mouse and human tissues

Yanxiang Deng[1,2], Marek Bartosovic[3], Sai Ma[4], Di Zhang[1], Petra Kukanja[3], Yang Xiao[5], Graham Su[1,2], Yang Liu[1,2], Xiaoyu Qin[1,2], Gorazd B. Rosoklija[6,7,8], Andrew J. Dwork[6,7,8,9], J. John Mann[6,7,10], Mina L. Xu[11], Stephanie Halene[2,12], Joseph E. Craft[13], Kam W. Leong[5,14], Maura Boldrini[6,7], Gonçalo Castelo-Branco[3,15,*], Rong Fan[1,2,11,16,*]

[1]Department of Biomedical Engineering, Yale University, New Haven, CT 06520, USA

[2]Yale Stem Cell Center and Yale Cancer Center, Yale School of Medicine, New Haven, CT 06520, USA

[3]Laboratory of Molecular Neurobiology, Department of Medical Biochemistry and Biophysics, Karolinska Institutet, Stockholm, Sweden

[4]Klarman Cell Observatory, Broad Institute of MIT and Harvard, Cambridge, MA 02142, USA

[5]Department of Biomedical Engineering, Columbia University, USA

[6]Department of Psychiatry, Columbia University, New York, NY, 10032 USA

[7]Division of Molecular Imaging and Neuropathology, New York State Psychiatric Institute, New York, NY, 10032 USA

[8]Macedonian Academy of Sciences & Arts, Skopje, Republic of Macedonia

[9]Department of Pathology and Cell Biology, Columbia University, New York, NY, 10032 USA

[10]Department of Radiology, Columbia University, New York, NY, 10032 USA

[11]Department of Pathology, Yale University School of Medicine, New Haven, CT, USA

[12]Section of Hematology, Department of Internal Medicine, and Yale Center for RNA Science and Medicine, Yale University School of Medicine, New Haven, CT, USA

[13]Department of Immunobiology, Yale University School of Medicine, New Haven, CT 06520, USA.

[14]Department of Systems Biology, Columbia University Irving Medical Center, New York, NY 10032, USA

[15]Ming Wai Lau Centre for Reparative Medicine, Stockholm node, Karolinska Institutet, Stockholm, Sweden

[16]Human and Translational Immunology Program, Yale School of Medicine, New Haven, CT 06520, USA

* Corresponding author. Email: rong.fan@yale.edu (R.F.) and goncalo.castelo-branco@ki.se (G.C.-B.)

Table of Contents

3 Supplementary Figures 1-12

15 Statistics and Reproducibility

16 Supplementary Tables 1-5 (captions included, tables in separate Excel file)

17 Supplementary References

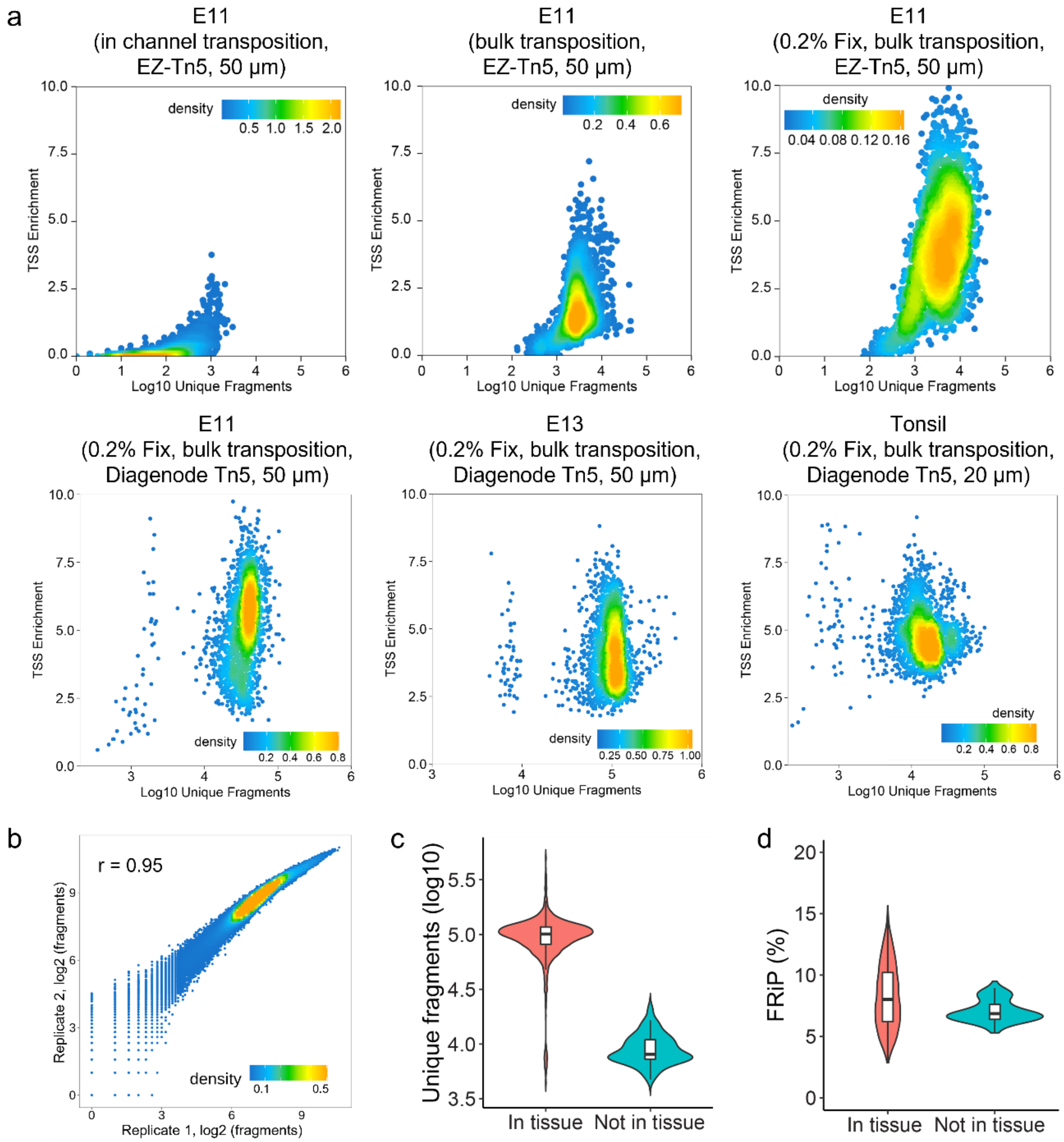

Supplementary Figure 1. Quality control metrics for spatial ATAC-seq datasets. a, Scatterplot showing the TSS enrichment score vs unique nuclear fragments per cell for different protocols and microfluidic channel width. **b,** Reproducibility between replicates on E13 mouse embryo. Correlation analysis of replicates was conducted by calculating the Pearson correlation coefficient of chromatin accessibility profiles between pseudo-bulk spatial-ATAC-seq data, which was constructed from the tile matrix in ArchR. Pearson correlation coefficient r = 0.95. **c,** Comparison of number of unique fragments in pixels in tissue (n = 2275) and not in tissue (n = 90) (E13 mouse embryo). **d,** Comparison of FRiP in pixels in tissue (n = 2275) and not in tissue (n = 90) (E13 mouse embryo) (The centers of the box plots are the median, the lower and upper hinges correspond to the first and third quartiles, and the whiskers show the 1.5 inter-quartile range).

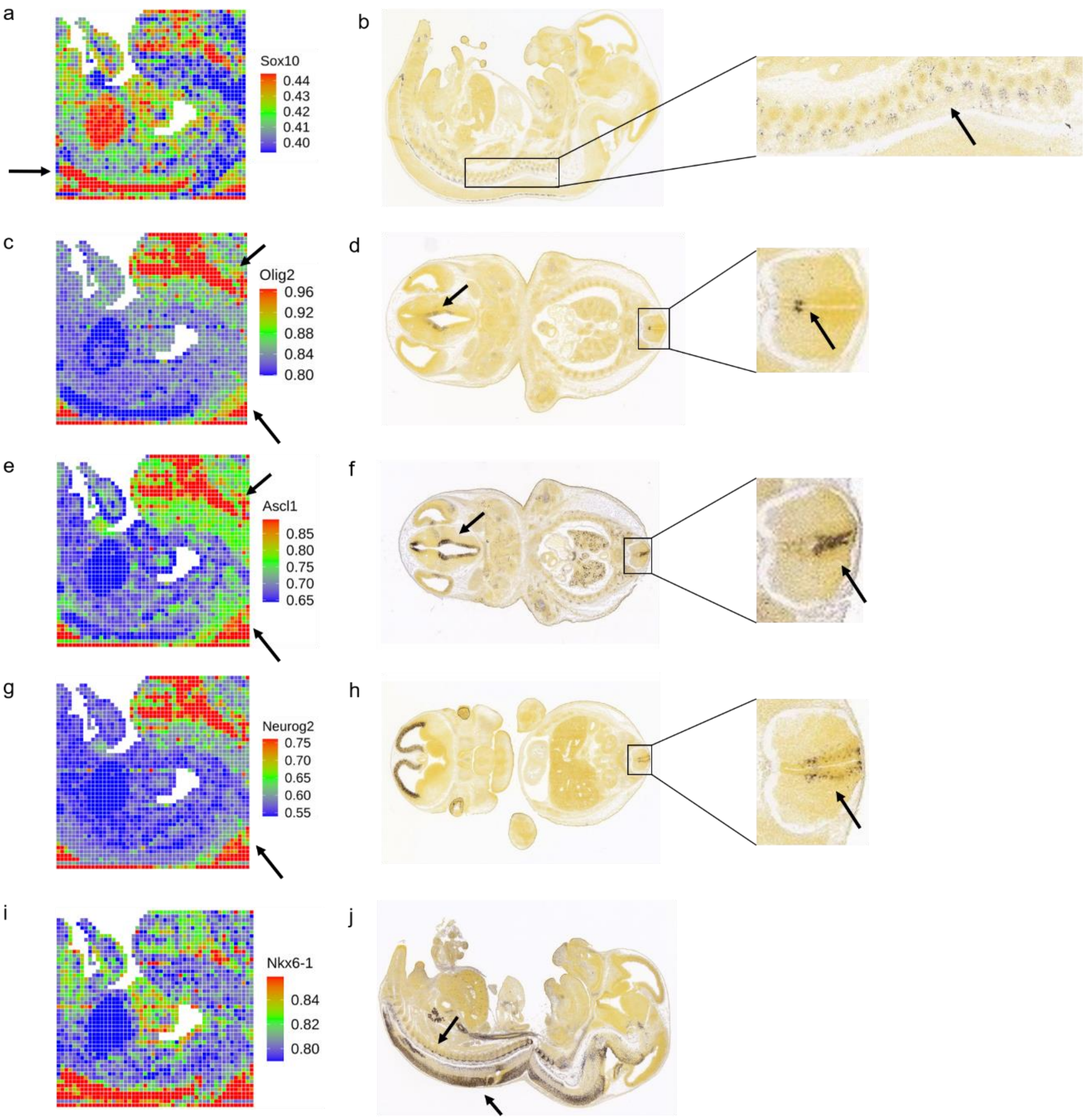

Supplementary Figure 2. Spatial mapping of gene scores in E13 mouse embryo and comparison with ISH reference data. a, c, e, g, i, Spatial mapping of the gene score for selected genes in E13 mouse embryo. **b, d, f, h, j,** In situ hybridization of selected genes at E13.5 mouse embryo from Allen Developing Mouse Brain Atlas[19].

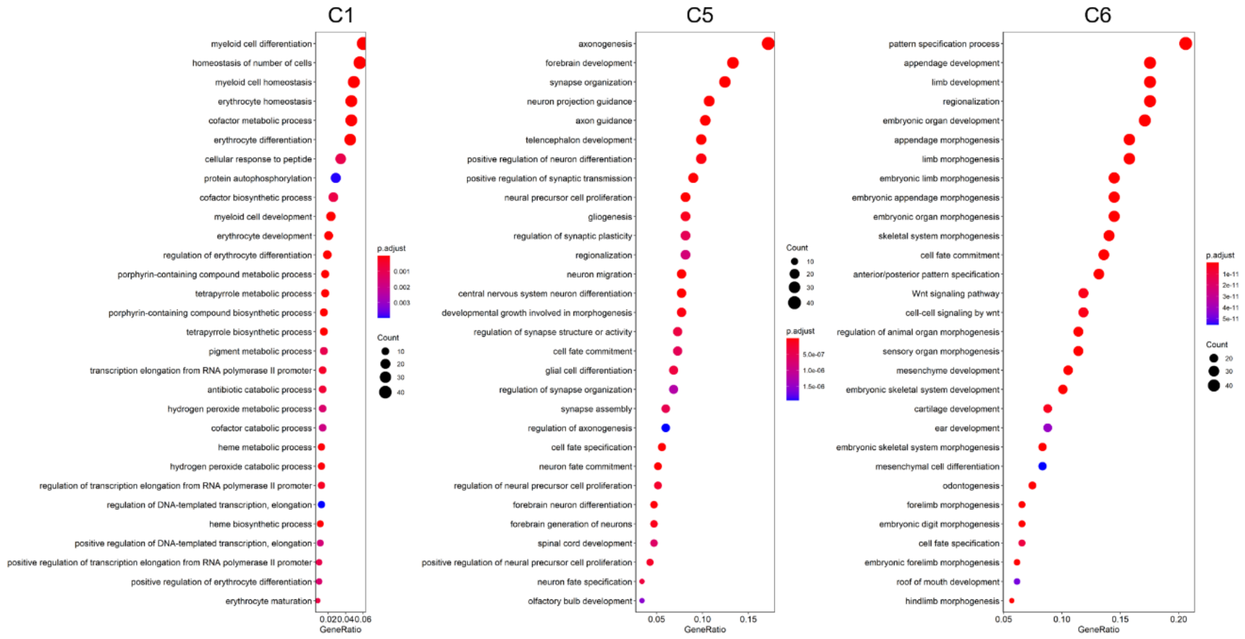

Supplementary Figure 3. GO enrichment analysis of spatial ATAC-seq data for E13 mouse embryo. GO enrichment analysis of differentially activated genes in selected clusters (C1, C5 and C6) (One-sided version of Fisher's exact test, p-value was adjusted for multiple comparisons by Benjamini & Hochberg method).

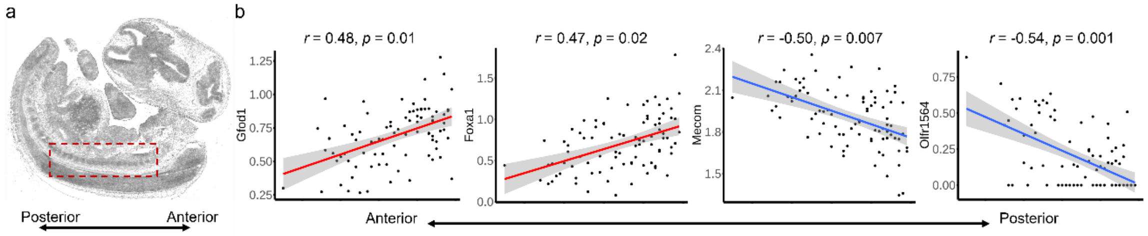

Supplementary Figure 4. Gene score along the anterior-posterior axis of the spine. a, Spine region of E13 mouse embryo profiled by spatial ATAC-seq. **b,** Selected genes found to form expression gradients along the anterior-posterior axis. The error bands are the 95% confidence level interval. (Correlation coefficient and p-value were calculated by the Pearson correlation method, and the p-value was adjusted for multiple comparisons by Benjamini & Hochberg method).

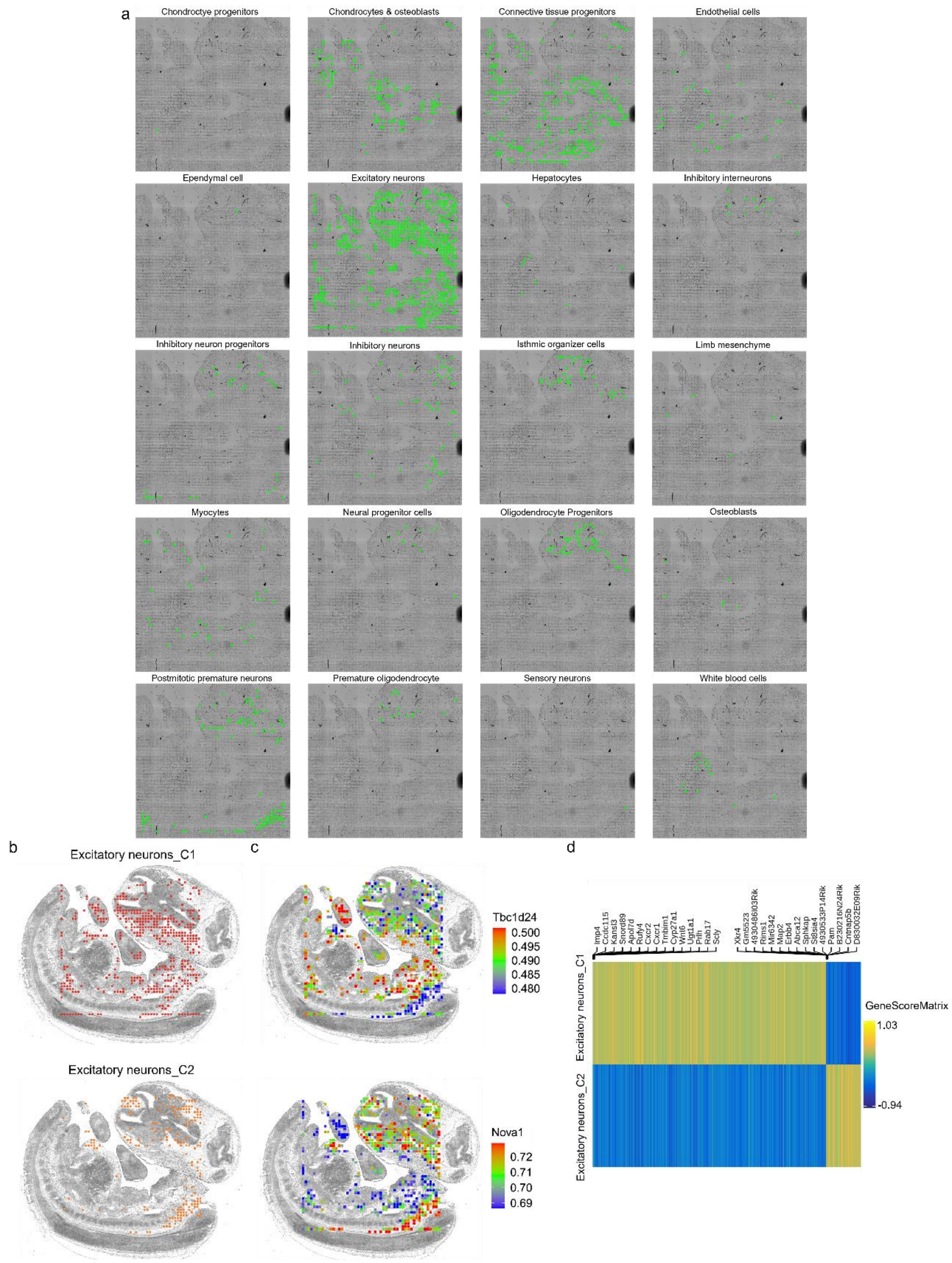

Supplementary Figure 5. Integrative analysis of spatial ATAC-seq and scRNA-seq for E13 mouse embryo and sub-clustering of excitatory neurons. a, Spatial mapping of cell types identified by label transfer from scRNA-seq[20] to spatial-ATAC-seq. **b-d,** refined clustering process enabled identification of sub-populations in excitatory neurons with distinct spatial distributions (**b**) and marker genes (**c, d**).

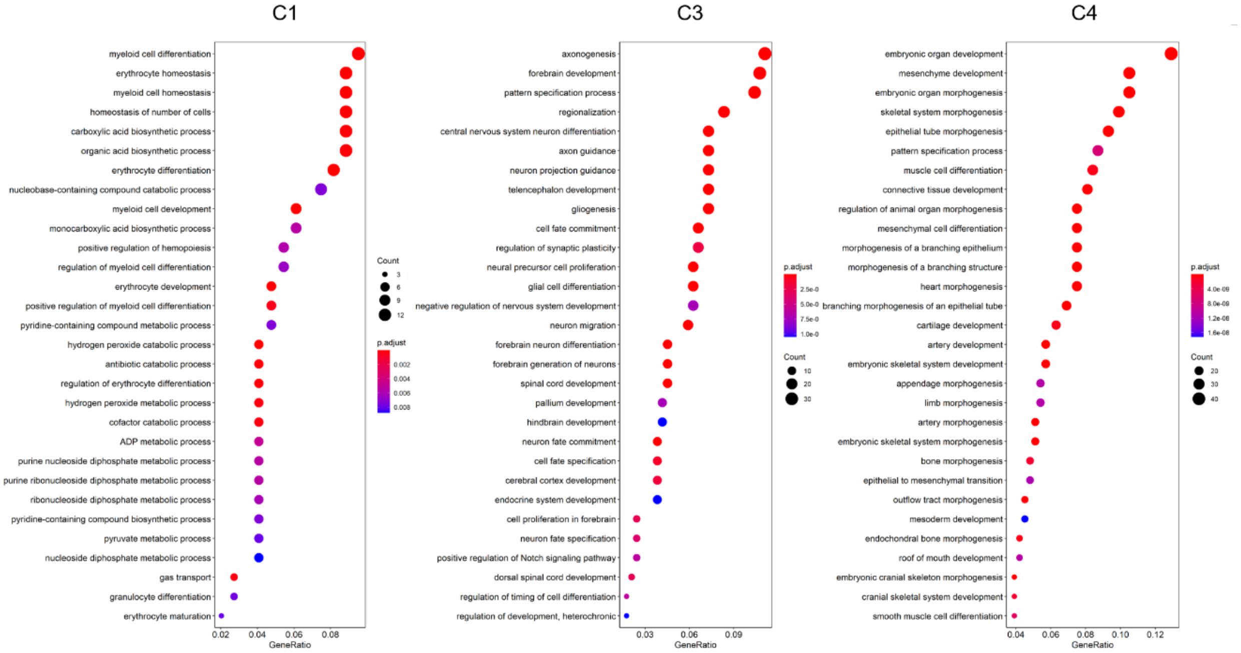

Supplementary Figure 6. GO enrichment analysis of spatial ATAC-seq data for E11 mouse embryo. GO enrichment analysis of differentially activated genes in selected clusters (C1, C3 and C4). (One-sided version of Fisher's exact test, p-value was adjusted for multiple comparisons by Benjamini & Hochberg method).

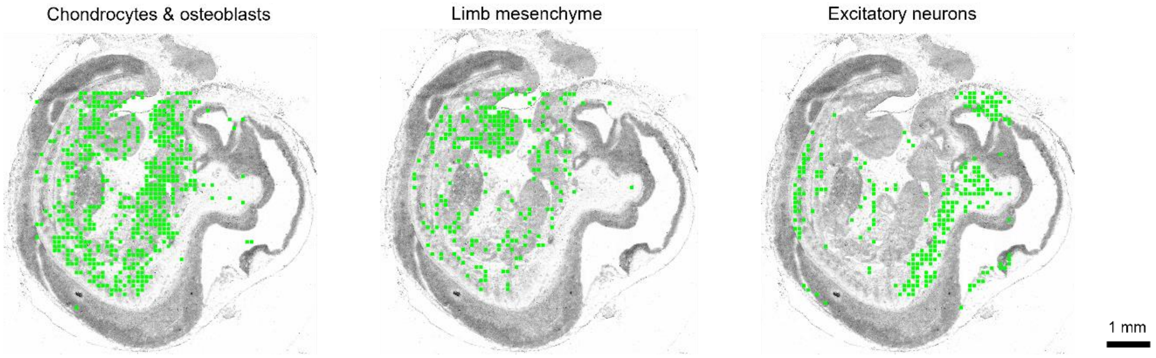

Supplementary Figure 7. Integrative analysis of spatial ATAC-seq and scRNA-seq for E11 mouse embryo and spatial map visualization of select cell types. Spatial mapping of selected cell types identified by label transferring from scRNA-seq to spatial-ATAC-seq.

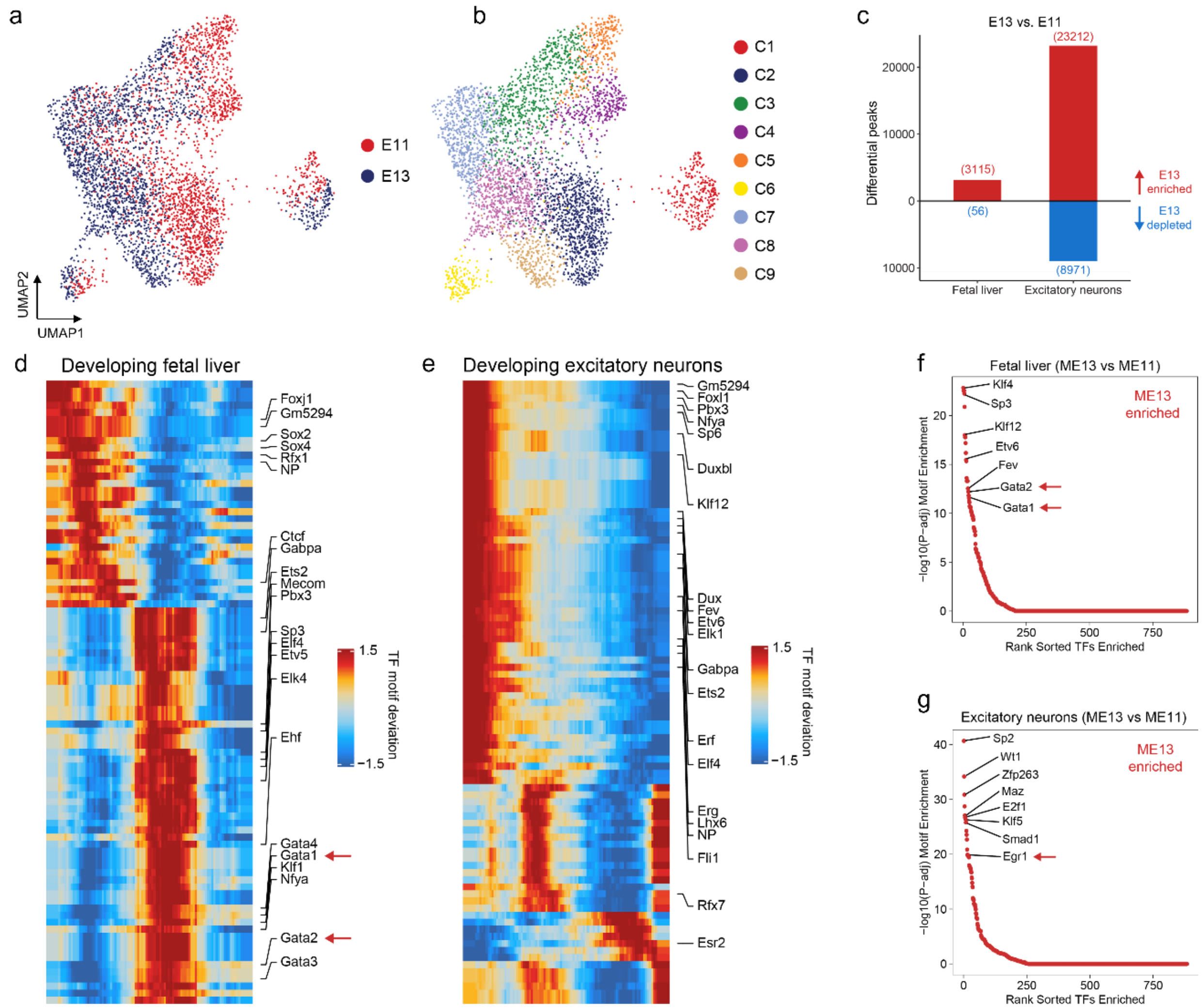

Supplementary Figure 8. Differential analysis of chromatin accessibility in E13 mouse embryo and E11 mouse embryo (50 μm pixel size). **a, b,** Co-embedding E13 mouse embryo and E11 mouse embryo spatial-ATAC-seq datasets, colored by sample **(a)** and ArchR identified clusters **(b)**. **c,** Differential peak analysis of E13 mouse embryo compared to E11 mouse embryo. **d,** Pseudo-time heatmap of TF motifs changes in the fetal liver from E11 to E13 mouse embryo. **e,** Pseudo-time heatmap of TF motifs changes in the excitatory neurons from E11 to E13 mouse embryo. **f,** Ranking of enriched motifs in the peaks that are more accessible in the fetal liver of E13 mouse embryo compared to E11 mouse embryo. **g,** Ranking of enriched motifs in the peaks that are more accessible in the excitatory neurons of E13 mouse embryo compared to E11 mouse embryo. (Hypergeometric test for motif enrichment, with FDR correction for multiple comparisons).

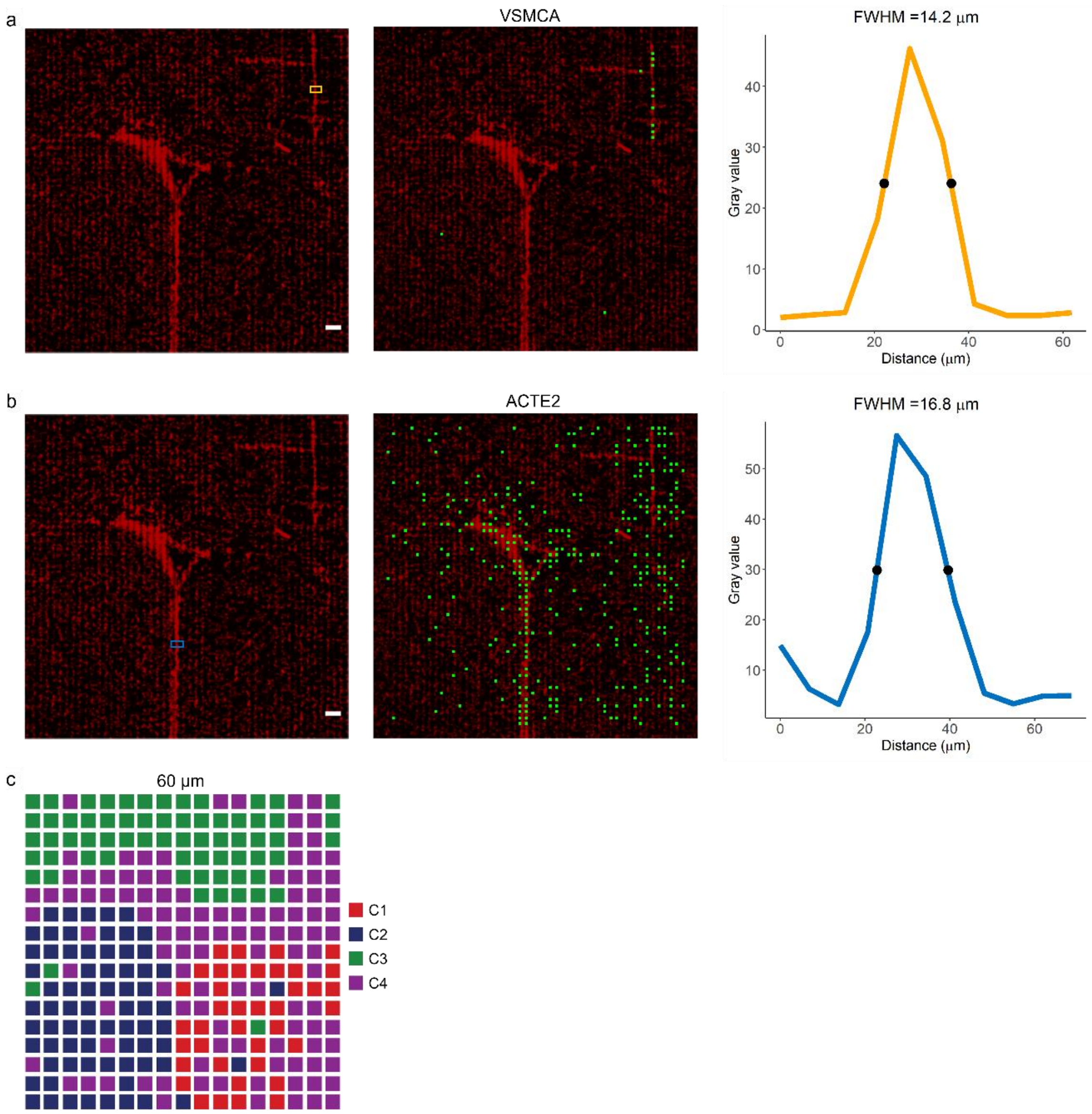

Supplementary Figure 9. Estimation of spatial-ATAC-seq resolution. a, b, Fluorescent image of nuclear staining in P21 mouse brain (left). Small box indicates region taken for line scans in Fiji ImageJ (v1.53q) [62] (right) and the cell type identified by label transfer from scRNA-seq[23] to spatial-ATAC-seq (20 µm pixel size) (middle). Full Width at Half Maximum (FWHM) was calculated as twice the distance between the peak and the halfmax (marked by black dots). Scale bars, 100 µm. Cell type annotation is in Supplementary Table 2. **c,** Unsupervised clustering analysis and spatial distribution of each cluster in binned spatial-ATAC-seq mouse brain data.

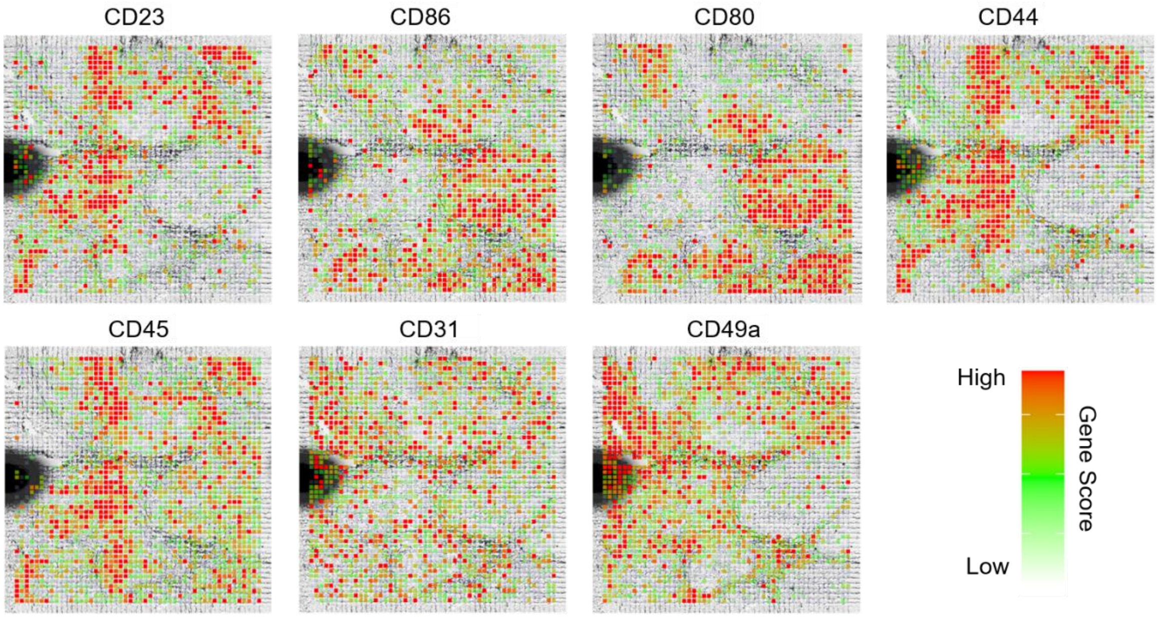

Supplementary Figure 10. Spatial chromatin accessibility mapping of human tonsil with 20 μm pixel size and visualization of specific marker genes. Spatial mapping of gene scores for selected genes.

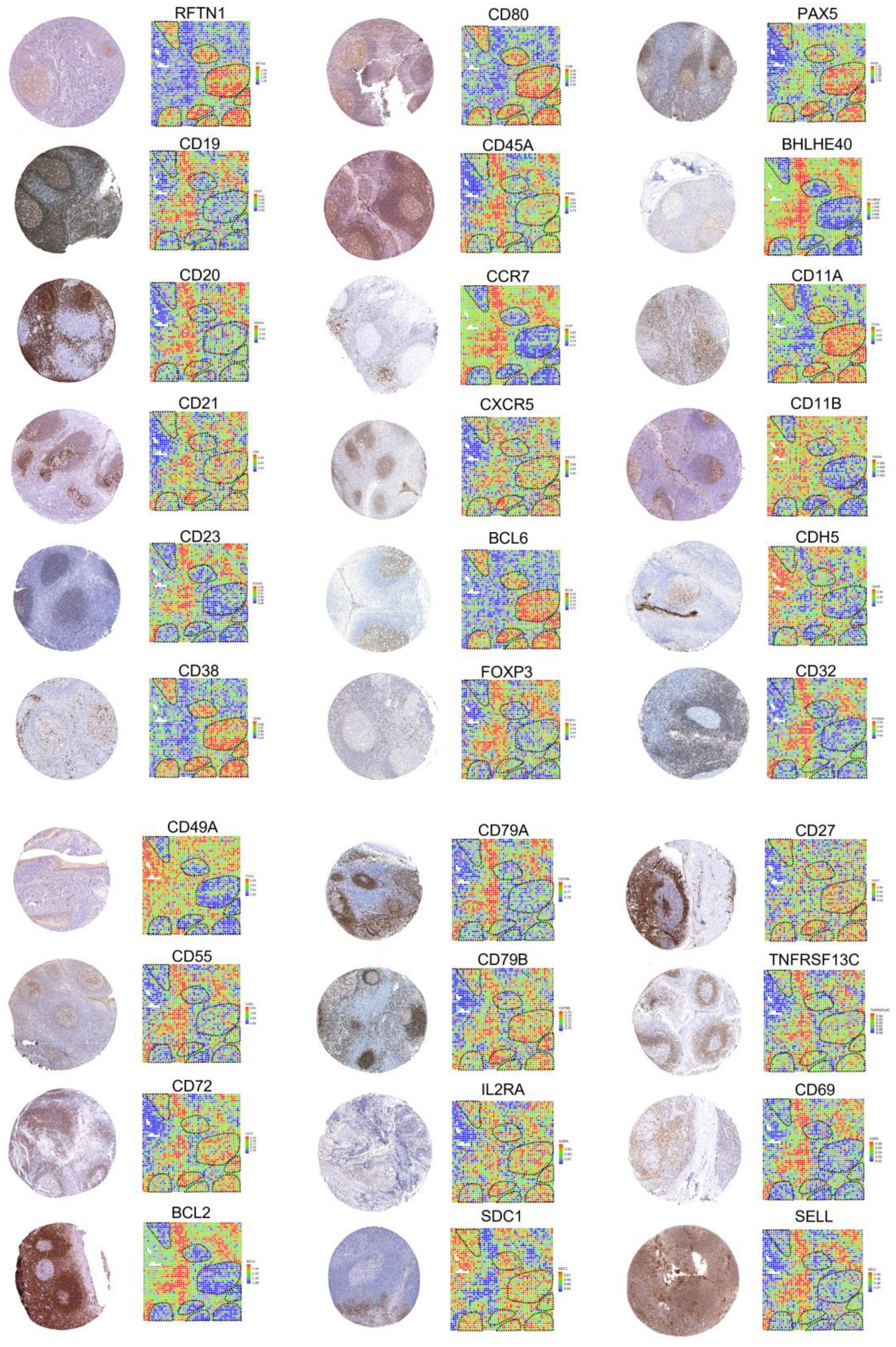

Supplementary Figure 11. Spatial chromatin accessibility gene score map in comparison with protein expression in human tonsil. The immunohistochemistry reference data were obtained from the Human Protein Atlas[63]**.**

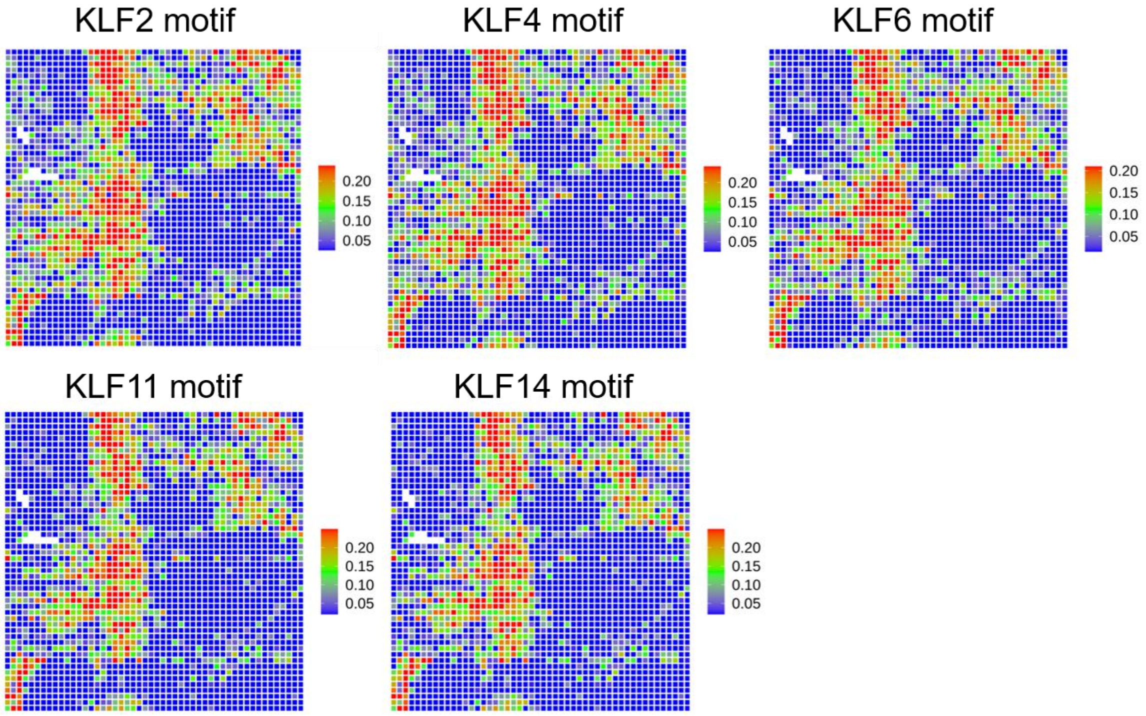

Supplementary Figure 12. Motif enrichment analysis of spatial-ATAC-seq data for human tonsil. Spatial mapping of motif deviation scores for KLF family transcription factors.

Statistics and Reproducibility

Figure	Description	# of times repeated
Fig.1c	Validation of *in situ* transposition and ligation using fluorescent DNA probes	1
Fig. 3a	Bright field image of mouse brain	1
Fig. 3b	Fluorescent image of nuclear staining in mouse brain	1
Fig. 3j	Fluorescent images of selected pixels containing single nucleus	1
Fig. 4a	H&E image of human tonsil	1
Extended Data Fig. 2a	H&E image of E13 mouse embryo	1
Extended Data Fig. 5a	H&E image of E11 mouse embryo	1
Supplementary Figure 9a,b	Fluorescent image of nuclear staining in mouse brain	1
Extended Data Fig. 9a	Nissl-stained image of human hippocampus	1

Supplementary Tables 1-5

Supplementary Table 1. Marker genes.

Supplementary Table 2. Cell type annotation in mouse brain.

Supplementary Table 3. DNA oligos used for PCR and preparation of sequencing library and DNA barcode sequences.

Supplementary Table 4. Chemicals and reagents.

Supplementary Table 5. Published data for data quality comparison and integrative data analysis

Supplementary References

62 Schindelin, J. *et al.* Fiji: an open-source platform for biological-image analysis. *Nature Methods* **9**, 676-682, doi:10.1038/nmeth.2019 (2012).

63 Uhlén, M. *et al.* Tissue-based map of the human proteome. *Science* **347**, 1260419, doi:10.1126/science.1260419 (2015).
