# Supplementary material for: Spatial profiling of chromatin accessibility in mouse and human tissues
Source: Nature. 2022 Aug 17;609(7926):375–83. doi: 10.1038/s41586-022-05094-1 (PMC9452302; doi:10.1038/s41586-022-05094-1)
Supplement: Supplementary file 2 — Reporting Summary [file 41586_2022_5094_MOESM2_ESM.pdf]

nature research

Corresponding author(s): Rong Fan, Gonçalo Castelo-Branco

Last updated by author(s): May 20, 2022

Reporting Summary

Nature Research wishes to improve the reproducibility of the work that we publish. This form provides structure for consistency and transparency in reporting. For further information on Nature Research policies, see our Editorial Policies and the Editorial Policy Checklist.

Statistics

For all statistical analyses, confirm that the following items are present in the figure legend, table legend, main text, or Methods section.

n/a	Confirmed	
☐	☒	The exact sample size (*n*) for each experimental group/condition, given as a discrete number and unit of measurement
☐	☒	A statement on whether measurements were taken from distinct samples or whether the same sample was measured repeatedly
☐	☒	The statistical test(s) used AND whether they are one- or two-sided<br>*Only common tests should be described solely by name; describe more complex techniques in the Methods section.*
☐	☒	A description of all covariates tested
☐	☒	A description of any assumptions or corrections, such as tests of normality and adjustment for multiple comparisons
☐	☒	A full description of the statistical parameters including central tendency (e.g. means) or other basic estimates (e.g. regression coefficient) AND variation (e.g. standard deviation) or associated estimates of uncertainty (e.g. confidence intervals)
☐	☒	For null hypothesis testing, the test statistic (e.g. *F*, *t*, *r*) with confidence intervals, effect sizes, degrees of freedom and *P* value noted<br>*Give P values as exact values whenever suitable.*
☒	☐	For Bayesian analysis, information on the choice of priors and Markov chain Monte Carlo settings
☒	☐	For hierarchical and complex designs, identification of the appropriate level for tests and full reporting of outcomes
☐	☒	Estimates of effect sizes (e.g. Cohen's *d*, Pearson's *r*), indicating how they were calculated

Our web collection on statistics for biologists contains articles on many of the points above.

Software and code

Policy information about availability of computer code

Data collection	EVOS FL Auto Software (REV 32044), Illumina HiSeq 4000 System, Illumina NovaSeq 6000 System.
Data analysis	R 3.6.1, python 3.7, ArchR 1.0.1, Seurat 3.2.3, clusterProfiler 3.16.1, GREAT 4.0.4, Cell Ranger ATAC v1.2, Snakemake v5.28.0, Adobe Illustrator v25.4.3, Fiji ImageJ 1.53q<br><br>Scripts for data analysis were written in R and python with code available at https://github.com/dyxmvp/Spatial_ATAC-seq and https://github.com/rongfan8/DBiT-seq

For manuscripts utilizing custom algorithms or software that are central to the research but not yet described in published literature, software must be made available to editors and reviewers. We strongly encourage code deposition in a community repository (e.g. GitHub). See the Nature Research guidelines for submitting code & software for further information.

Data

Policy information about availability of data

All manuscripts must include a data availability statement. This statement should provide the following information, where applicable:

- Accession codes, unique identifiers, or web links for publicly available datasets
- A list of figures that have associated raw data
- A description of any restrictions on data availability

Raw and processed data reported in this paper are deposited in the Gene Expression Omnibus (GEO) with accession code GSE171943. Resulting fastq files were aligned to the mouse reference genome (mm10) or human reference genome(GRCh38). Published data for data quality comparison and integrative data analysis include Flash frozen cortex, hippocampus, and ventricular zone from embryonic mouse brain (E18) (https://www.10xgenomics.com/resources/datasets/flash-frozen-cortex-hippocampus-and-ventricular-zone-from-embryonic-mouse-brain-e-18-1-standard-1-2-0), ENCODE mouse embryo ATAC-seq (11.5 days) (https://

www.encodeproject.org/search/?type=Experiment&status=released&related_series.@type=OrganismDevelopmentSeries&replicates.library.biosample.organism.scientific_name=Mus+musculus&assay_title=ATAC-seq&life_stage_age=embryonic%2011.5%20days), ENCODE mouse embryo ATAC-seq (13.5 days) (https://www.encodeproject.org/search/?type=Experiment&status=released&related_series.@type=OrganismDevelopmentSeries&replicates.library.biosample.organism.scientific_name=Mus+musculus&assay_title=ATAC-seq&life_stage_age=embryonic%2013.5%20days), Mouse organogenesis cell atlas (MOCA) (https://oncoscape.v3.sttrcancer.org/atlas.gs.washington.edu.mouse.rna/downloads), Atlas of gene regulatory elements in adult mouse cerebrum (http://catlas.org/mousebrain/#!/downloads), Atlas of the Adolescent Mouse Brain (http://mousebrain.org/adolescent/downloads.html), Human hippocampus scATAC-seq data (GSE147672), Human tonsil scATAC-seq data (GSE165860), Human tonsil scRNA-seq data (GSE165860), and Allen Developing Mouse Brain Atlas (https://developingmouse.brain-map.org/).

Field-specific reporting

Please select the one below that is the best fit for your research. If you are not sure, read the appropriate sections before making your selection.

☒ Life sciences ☐ Behavioural & social sciences ☐ Ecological, evolutionary & environmental sciences

For a reference copy of the document with all sections, see nature.com/documents/nr-reporting-summary-flat.pdf

Life sciences study design

All studies must disclose on these points even when the disclosure is negative.

Sample size	No directly relevant. No sample size calculation was performed. Samples sizes were chosen primarily based on experiment length, sample availability, and sequencing costs. The current manuscript mainly described a new method for profiling spatially resolved chromatin accessibility and the sample sizes are sufficient because each sample serves as a proof-of-concept for the new technology.
Data exclusions	No data were excluded from the study.
Replication	All attempts at replication was successful. For E13 mouse embryo, two replicates have been done on adjacent tissue sections to test the reproducibility of the new technology. Other experiments were performed once to serve as a proof-of-concept for the new technology.
Randomization	Randomization was not applicable because the focus of this paper is the development of a new method for profiling spatially resolved chromatin accessibility and did not involve allocating samples/organisms/participants into experimental groups.
Blinding	Blinding was not applicable because the focus of this paper is the development of a new method for profiling spatially resolved chromatin accessibility and did not involve group allocation, and by extension, blinding.

Reporting for specific materials, systems and methods

We require information from authors about some types of materials, experimental systems and methods used in many studies. Here, indicate whether each material, system or method listed is relevant to your study. If you are not sure if a list item applies to your research, read the appropriate section before selecting a response.

Materials & experimental systems

n/a	Involved in the study	
☒	☐	Antibodies
☐	☒	Eukaryotic cell lines
☒	☐	Palaeontology and archaeology
☐	☒	Animals and other organisms
☐	☒	Human research participants
☒	☐	Clinical data
☒	☐	Dual use research of concern

Methods

n/a	Involved in the study	
☒	☐	ChIP-seq
☒	☐	Flow cytometry
☒	☐	MRI-based neuroimaging

Eukaryotic cell lines

Policy information about cell lines

Cell line source(s)	NIH/3T3 cells were from ATCC.
Authentication	None of the cell lines were authenticated.
Mycoplasma contamination	Cell lines were not tested for Mycoplasma contamination.
Commonly misidentified lines (See ICLAC register)	No commonly misidentified cell lines were used.

Animals and other organisms

Policy information about studies involving animals; ARRIVE guidelines recommended for reporting animal research

Laboratory animals	The mouse line Sox10:Cre-RCE:LoxP (EGFP), on a C57BL/6xCD1 mixed genetic background, female was used for experiments on P21 mice.<br><br>Mice were kept in individually ventilated cages (IVC sealsafe GM500, Tecniplast) with hardwood bedding, nesting material, shredded paper, gnawing sticks and card box shelter. Cages were changed every other week. Mice received regular chew diet (either R70 diet or R34, Lantmännen Lantbruk, Sweden) and water using a water bottle that was changed weekly. Housing parameters including relative humidity, temperature, and ventilation were established following the European convention for the protection of vertebrate animals used for experimental and other scientific purposes treaty ETS 123. Specifically, consistent relative air humidity and temperature were set to 50% and 22°C, and the air quality was controlled with the use of stand-alone air handling units supplemented with HEPA filter. Monitoring of husbandry parameters is done using ScanClime (Scanbur) units. The following light/dark cycle was used: dawn 6:00–7:00, daylight 7:00–18:00, dusk 18:00–19:00, night 19:00–6:00.
Wild animals	No wild animals were used in the study.
Field-collected samples	No field collected samples were used in the study.
Ethics oversight	Experimental procedures on juvenile (P21) mice were conducted in accordance with the European directive 2010/63/EU, local Swedish directive L150/SJVFS/2019:9, Saknr L150 and Karolinska Institutet complementary guidelines for procurement and use of laboratory animals, Dnr 1937/03-640. The procedures described here were approved by Stockholms Norra Djurförsöksetiska nämnd, the local committee for ethical experiments on laboratory animals in Sweden, lic.nr. 1995/2019 and 7029/2020.

Note that full information on the approval of the study protocol must also be provided in the manuscript.

Human research participants

Policy information about studies involving human research participants

Population characteristics	Age: 31, Gender: Male. The subject was free of neuropsychiatric illness, had clear neuropathological exam, negative brain toxicology for psychoactive drugs, medication and alcohol.
Recruitment	The subject selected was free of neuropsychiatric illness based on our validated psychological autopsy interview of the next of kin, died of sudden death (industrial accident) with short agonal state (that can affect brain oxygenation if prolonged), had short post-mortem interval (6.5 Hour), clear neuropathological exam, negative brain toxicology for psychoactive drugs, medication and alcohol, and good RNA quality (RNA integrity number 8.50). Biases were not applicable because the focus of this paper is the development of a new method for profiling spatially resolved chromatin accessibility and each sample serves as a proof-of-concept for the new technology.
Ethics oversight	All procedures of brain collection and autopsy were conducted with Institutional Review Board approvals and informed consent from the next of kin.

Note that full information on the approval of the study protocol must also be provided in the manuscript.
